# Mapping of the Receptive Fields in the Optic Tectum of Chicken (*Gallus gallus*) Using Sparse Noise

**DOI:** 10.1371/journal.pone.0060782

**Published:** 2013-04-08

**Authors:** Josine Verhaal, Harald Luksch

**Affiliations:** Lehrstuhl für Zoologie, Technische Universität München, Freising-Weihenstephan, Germany; Universidade Federal do ABC, Brazil

## Abstract

The optic tectum plays a key role in visual processing in birds. While the input from the retina is topographic in the superficial layers, the deep layers project to the thalamic nucleus rotundus in a functional topographical manner. Although the receptive fields of tectal neurons in birds have been mapped before, a high resolution description of the white and black subfields of the receptive fields of tectal neurons is not available. We measured the receptive fields of neurons in the different layers of the tectum of anesthetized chickens with black and white stimuli that were flashed on a grey background in fast progression. Our results show that neurons in the deep layers of the optic tectum tend to respond stronger to black stimuli compared to white stimuli. In addition, the receptive field sizes are larger when measured using black stimuli than with white stimuli. While the black subfield was significantly larger than the white subfield for the intermediate and deep layers, no significant effects were found for the superficial layers. Finally, we investigated the optimal stimulus size in a subset of the neurons and found that these cells respond best to small white stimuli and to large black stimuli. In the majority of the cases the response was stronger to a large black bar than to a small white bar. We propose that such a stronger response to black stimuli might be advantageous for the detection of darker objects against the brighter sky.

## Introduction

The optic tectum or its mammalian counterpart the superior colliculus is a key structure for processing visual information [1], [2]. Its primary role is to elicit orienting responses to stimuli in the sensory surround of the animal in a bottom-up fashion.

The optic tectum contains 15 layers [3], which can be grouped into the superficial layers (layers 1–7), the intermediate layers (layers 8–12) and the deep layers (layers 13–15). While the superficial layers of the optic tectum receive visual information from every point in space in a map-like fashion [4], [5], its projection to the thalamic nucleus rotundus carries information about stimulus properties such as its color or movement [6], [7], [8].

The spatial receptive field is classically defined as the area of space within which the discharge of a neuron can be modulated. The receptive fields of tectal neurons seem to possess a center-surround organization [9], [10], [11], [12], [13], [14] where neurons increase their activity in response to stimuli in the excitatory center while being inhibited by stimuli within the inhibitory surround [14], [15], [16], [17].

The receptive field increases in size and its shape becomes more complex from the superficial to the deep layers [18]. While the excitatory centers of the receptive fields of neurons in the superficial layers are only a few degrees in size, they can span up large parts of the visual space for neurons in the deeper layers [9], [19].

While the receptive fields of tectal neurons in birds have been mapped before, these studies were often restricted to one kind of contrast, e.g. black or white [5], [20], [21]. Moreover, receptive fields have been mapped by moving stimuli through the receptive field [9], [12], [22], [23], [24], which results in a rough estimation of the borders of the receptive field only. Since neurons in the visual cortex have receptive fields with subfields that respond differentially to an increase and decrease in luminance [25], [26], receptive fields should be mapped using black and white stimuli.

**Figure 1:**
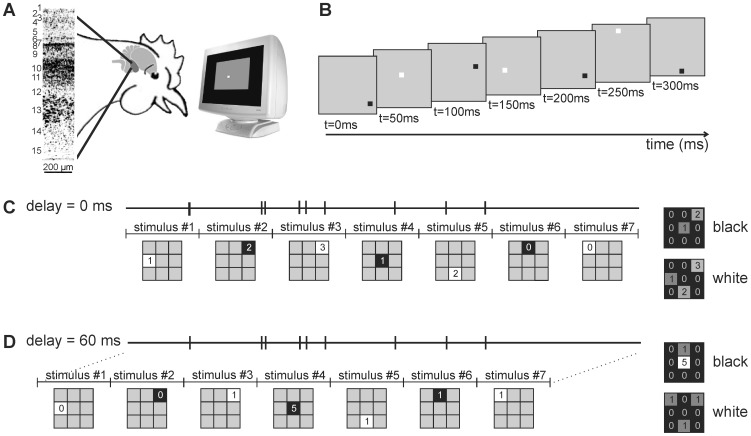
Method. a) Extracellular recordings were made from the superficial, intermediate and deep layers of the optic tectum in chickens while the contralateral eye was stimulated with white and black stimuli presented on a CRT monitor. b) Black and white stimuli on a grey background were shown in fast progression (see methods for details). c) Reverse correlation method. For each spike we looked which stimulus was presented at that time (for delay = 0 ms) and we increased the spike count for the stimulus presented at this position. The result is an array showing the number of spikes evoked by the stimulus presented at the moment that the spikes occurred. d) Next we looked which stimulus was present a short delay before the occurrence of the spikes (delay = 60 ms). For simplicity the stimulus grid is reduced to 3×3 positions and data arrays are only shown for delay = 0 and delay = 60 ms. However, analyses were done for stimuli occurring 0–200 ms before in 5 ms steps (for stimulus duration of 50 ms).

This study investigates the receptive field properties of neurons in the superficial, intermediate and deep layers of the optic tectum in chicken. In order to map the receptive field precisely, we applied a reverse correlation method using sparse-noise stimuli (see figure 1), which was introduced for the visual cortex [26], [27], [28], [29]. To the best of our knowledge, this is the first application of this method to the optic tectum in chicken. Our results show that most units respond stronger to black stimuli than to white stimuli. Accordingly, the black subfield is larger than the white subfield and this difference increases along the depth of the tectum. Interestingly, for those neurons tested with both black and white moving bars, the most effective white stimulus was often very small while the best black stimulus was always large.

## Materials and Methods

### Animals

Thirty-one chickens (*Gallus gallus*) of 4–6 weeks of age were used for this study. The animals were bred in-house and were kept in small groups on a 12 h dark-light cycle. From a few days of age the animals had access to sand baths as cage enrichment. The animals had ad libitum access to water and food.

### Ethic Statement

All experiments were performed according to the principles regarding the care and use of animals adopted by the German Animal Welfare Law for the prevention of cruelty to animals. The study was approved by the Government of Upper Bavaria, Germany. All surgical procedures and recordings were done under Ketamine/Xylazine anesthesia and all efforts were made to minimize suffering.

### Surgery

The animals were anesthetized using Ketamine/Xylazine i.m. (40 mg/kg Inresa, Freiburg, Germany/12 mg/kg Bayer, Leverkusen, Germany). Additional doses were given when necessary for the duration of the surgery. Approximately 30 minutes before the end of the surgery a pump (univentor 801 syringe pump, TSE systems, Zejtun, Malta) was installed which infused Ketamine/Xylazine (20 mg/h/kg/6 mg/h/kg i.m.) for the rest of the experiment.

The feathers on the head were trimmed and the auditory cavities were locally anesthetized with lidocaine (AstraZeneca, Wedel, Germany). The anesthetized chickens were placed on a regulated temperature pad. Their head was fixed in a customized stereotactic head holder, which did not obscure the visual field. The head was positioned in the standard position with the beak at 45 degrees relative to the ears. We entered the tectum using a lateral approach. Right above the accessible tectum the skull was opened and a small hole was made into the dura mater through which the electrode could enter the brain. In addition, the contralateral eye was fixated in the open position. Since the ipsilateral eye was closed and recordings were performed in a darkened room, we did not take any additional measure to cover the ipsilateral eye.

### Recordings

Recordings were made from the 'accessible tectum' which responds to stimuli presented at 60 degrees in the lateral part of the visual field. Several penetrations were made in each animal, whereby the entry point of the electrode was slightly varied in order to assure recordings from different neurons. A single electrode was lowered under an angle perpendicular to the brain surface using a computerized micromanipulator (Omnidrive, NeuroStar, Germany). Neuronal activity was recorded with an electrode amplifier (RZ5, Tucker-Davis Technologies, Alachua, USA) using Tungsten electrodes with an impedance of ∼2 MΩ (Alpha Omega, Nazareth, Israel). Signals were amplified, online bandpass-filtered between 300–3000 Hz, and monitored on an oscilloscope and as an audio signal through headphones.

The threshold for detecting spikes was set at a 3-to-1 signal-to-noise ratio. Signals that passed this threshold were stored as waveforms and time point events. Both the recorded data trace and the waveforms were sampled at 25 kHz and stored for offline analysis.

The neurons were assigned to the superficial, intermediate or deep layers based on the depth reading of the micromanipulator. We never recorded any neurons above 180 µm in order to avoid recordings from retinal ganglion cell afferents. Neurons recorded between 180 and 500 µm were regarded as belonging to the superficial layers. Neurons recorded between 500 and 1200 µm were regarded as belonging to the intermediate layers, while those recorded deeper than 1200 µm were regarded as deep layer neurons.

### Visual Stimulation

Before recording, the animal itself was rotated so that the lateral fovea of the eye [30], [31] contralateral to the exposed tectum was facing the monitor. The stimuli were presented on a high-end CRT monitor (Iiyama 514, Nagaro, Japan) operating at 200 Hz, far above the flicker frequency of chickens [32]. The monitor was located at ∼33 cm from the eye.

Stimuli were programmed using the VisionEgg toolbox which allows frame-by-frame control of the visual stimuli [33]. The monitor was encapsulated in a Faraday cage except for the screen. In front of the screen a transparent foil (ProtectES-HF, MB Abschirmungstechnik, Kaufingen, Germany) was put to prevent high-frequency noise from the monitor. Luminance of the white stimulus (colorcode #FFFFFF; 360 lx) and black stimulus (colorcode #000000; 2 lx) were measured at the middle of the screen but outside of the protective foil. The grey value for the background color was selected to be halfway black and white (colorcode #A1A1A1). The monitor was turned on at the beginning of the recording day so that it had >1 hour to warm up.

### Receptive Field Approximation

In order to allow detailed mapping of the receptive field it is important to present the stimuli in a part of the visual field that is neither too large nor too small. If the grid is too large, the individual grid units are too large to obtain a detailed receptive field. However, if the grid is too small, only part of the receptive field may be covered. Therefore we made an initial rough estimation of the receptive field by moving a bar stimulus over the screen using a computer mouse. The size and contrast of the bar (white, black or grey) as well as the background (white, black or grey) could be adapted during the search process. The experimenter roughly outlined the borders of the receptive field by manually moving white and black bar stimuli while listening to the audio signal on headphones. The positions of the moving bar at times that spikes occurred were plotted on the screen. While this method does not take into account the latency of the cells in response to visual stimulation, it gives a fairly good estimation of the receptive field, since the bar was not moved very rapidly.

Once the outline was determined, the experimenter specified the grid in which the stimuli were to be presented. This grid was determined so that it was approximately square and extended roughly 10% beyond the receptive field outline estimated before on either side. The grid was divided into either 15×15 units or 20×20 units (see Receptive field determination for details); the exact size of a grid unit was stored in order to determine the size of the receptive field in the offline analysis.

Since the receptive fields for neurons in intermediate and deeper layers were larger than those in the superficial layers the grid was larger for those cells. Each grid unit was 1.5+/−0.9SD degrees wide by 1.5+/−0.8SD degrees tall for neurons recorded from the superficial layers, 2.6+/−1.1SD degrees wide by 2.5+/−0.8SD degrees tall for intermediate layers, and 3.1+/−0.9SD degrees wide by 2.9+/−0.7SD tall for the deep layers.

### Receptive Field Determination

We presented stationary white and black stimuli with the size of one grid unit on a grey background without inter-trial-interval. The duration of each stimulus was 50 ms or 100 ms. As our first recordings with 50 ms stimuli elicited comparable responses to 100 ms stimuli, we switched to stimulus sequences containing 50 ms stimuli. The grid was initially set to 15×15 stimuli. Later we increased the resolution of the grid to 20×20 units in order to increase spatial resolution. Therefore, each recording consisted of 20 trials during which 450 (15×15×2 contrasts) or 800 (20×20×2 contrasts) stimuli were shown, resulting in a total of 9000 (450×20 trials) or 16000 (800×20 trials) stimulus presentations. In each trial, stimulus presentation was randomized so that the stimuli were presented in a different order.

**Figure 2:**
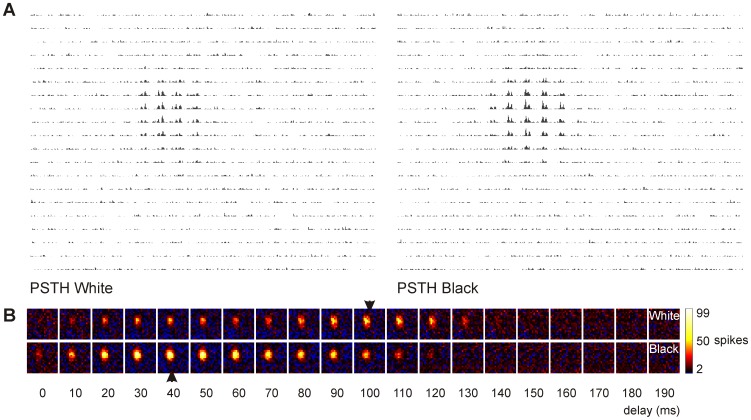
Receptive field of a neuron using PSTH and reverse correlation. a) PSTH of a single unit response recorded at a depth of 197 µm (superficial layers) in response to white and black stimuli on a grey background for each grid position on a 20×20 axis. In this specific case each grid position covered 0.3×0.3 degrees of visual field. For plotting purpose the axes of the PSTH were discarded. b) Receptive field of the same neuron where every spike is reversely correlated to the white (upper row) and black (lower row) stimulus shown a short period before (delay = 0–190 ms). The arrows show the optimal latency for which the highest autocorrelation was found for black and white. The white subfield is 1.34 degrees (at delay = 100 ms) and black subfield is 1.53 degrees (at delay = 40 ms). The maximum response evoked to stimulation at one grid position in this particular example was 99 spikes.

**Figure 3:**
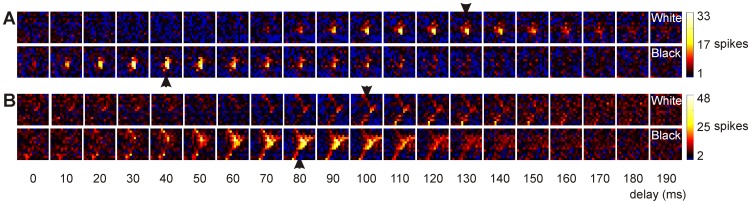
Receptive fields of two neurons in the optic tectum. a) Receptive field of a neuron recorded at 1210 µm presented as the response to white and black stimuli shown 0–190 ms before. The neuron responded with a short delay to black (40 ms) and with a longer delay to white (130 ms) and therefore could be responsive to decrease in luminance. In this particular case, the stimuli were shown on 15×15 positions which each covered 3.4×3.0 degrees of the visual field. The white subfield (at delay = 130 ms) was 10.8 degrees in size while the black subfield was 14.9 degrees (at delay = 40 ms). b) Receptive field of a neuron recorded at 1090 µm. In this particular case, the stimuli were shown on 15×15 positions which each covered 4.2×3.5 degrees of visual field. Please notice that the black subfield is larger than the white subfield (for this neuron the white subfield was 11.1 degrees in size while the black subfield was 19.4 degrees).

**Figure 4:**
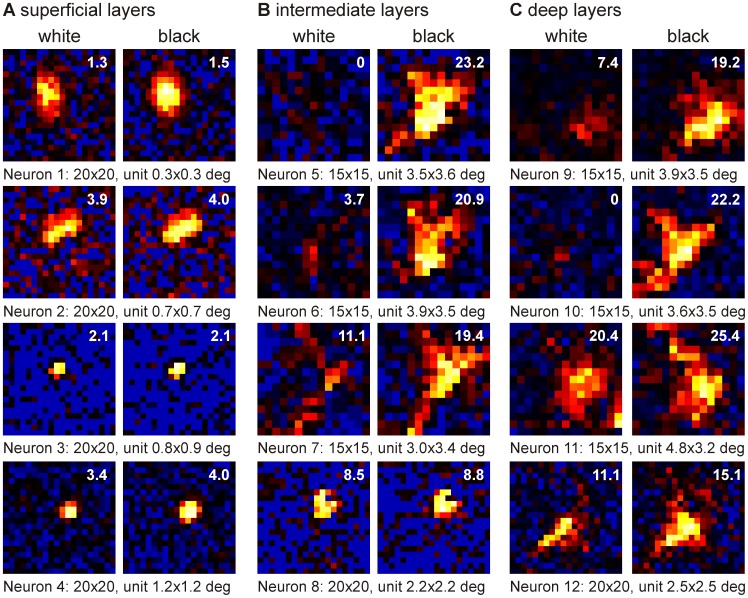
White and black subfields of the receptive fields. Examples of the white and black subfields of the receptive fields of twelve neurons at their optimal latency recorded in the a) superficial layers (neuron 1–4), b) intermediate layers (neuron 5–8) and c) deep layers (neuron 9–12). Below the picture is the information regarding the number of grid units (15×15 or 20×20) as well as the size of each grid unit in degrees (width×height). The calculated size of the subfields is depicted in the upper right corner of each picture.

After the recording was finished, the occurrence of the spikes was reversely correlated with the visual stimulus [26], [29]. Therefore, whenever a spike occurred, we looked back in time which stimulus was presented (see figure 1). The number of spikes was then plotted for stimuli presented 0–200 ms (for stimulus duration of 50 ms) or 0–400 ms (for stimulus duration of 100 ms) before (called “delay” in the rest of the manuscript) in 5 ms steps. This resulted in several arrays containing the number of spikes for a stimulus shown several milliseconds before. These arrays of counts (“grid pixels”) are then color-coded using a customized colormap. Black represents the spontaneous activity. Activity above baseline is shown in a heat colormap ranging from black to white via red and yellow. Activity below baseline ranges from black (baseline) to blue (0 spikes). No interpolation between the pixels was performed. This results in the receptive fields as shown in figures 2, 3 and 4.

The latency of the response, and its corresponding receptive field, was determined using the autocorrelation between each pixel and the sum of its surrounding pixels. Since the correlation takes into account the mean and variance across the array, high autocorrelation scores are only obtained when high-activity pixels cluster together in a receptive field (i.e. when pixels with high spike counts are grouped together). This autocorrelation was computed separately for each possible delay and the delay with the maximum autocorrelation was chosen as the optimal latency. The optimal latency (and the corresponding receptive field) was calculated independently for white and for black stimuli.

### Responses to Moving Stimuli

A subset of the neurons was tested with either white or black bars moving across the screen. The stimulus consisted of a white bar on black background or a black bar on a grey background. The bars moved through the receptive field at 0, 45, 90, 135, 180, 225, 270 or 315 degrees. The bars had a 2∶1 width:length ratio with a width of 0.5, 1, 2 or 4 degrees.

The bars were swept through the center of the receptive fields, starting and ending outside the receptive field with a speed of 10, 20 or 40 degrees/s. We defined the middle and the borders of the receptive field online based on the evoked spikes. The stimuli were swept from 2 grid units outside the receptive field till 2 grid units past the border of the receptive field. The best stimulus was defined as the condition in which the largest response was evoked, defined as the highest number of spikes within any 200 ms window.

We estimated the properties by moving the bars through the receptive field, thereby varying direction, size or speed. First a 1 degree bar was swapped through the receptive field on 8 different trajectories, covering all directions with a 45 degrees raster. Next we kept the trajectory at the best direction (defined as the best peak response over a 200 ms period) and varied the size of the stimulus. Finally we varied the speed of the bar moving through the receptive field moving in the best direction using a bar size evoking the best response. The inter-trial-interval was 2 seconds for each of these procedures.

### Histology

At the end of the experiment, a lesion was set by passing a 20 µA DC current of 10 seconds duration through the electrode to mark the final recording position. After 15 min, the chicken was deeply anesthetized with an additional dose of pentobarbital (200 mg/kg i.p.) and perfused transcardially with 0.1 M phosphate-buffered saline followed by 4% paraformaldehyde solution in 0.1 M phosphate buffer. After cryoprotection, brains were cut at 75 µm in the frontal plane, stained with Cresyl violet, and microscopically analyzed to reconstruct recording positions from the lesions.

### Analysis

The data was imported into Open Electrophysiology [34] for offline sorting. Using principle component analysis the spikes were grouped into clusters of spikes based on the three features that could explain the highest variance in their waveforms. Whether or not the spikes in these clusters were regarded as coming from a single cell was assessed for each cluster separately on the basis of their spike shape and the inter-spike interval. In case one or more single unit clusters were identified, any additional spikes not belonging to any of these clusters were discarded. While regularly spiking neurons in the deeper layers could be easily isolated and sorted into single unit responses (SU), the spikes of bursty units in the superficial layers were often more difficult to isolate. We often observed high-frequency bursts of spikes which resemble the tectal bursts observed in pigeons [35]. Due to this high-frequency bursting, it was often impossible to determine whether the spikes recorded from a bursty unit came from a single or multiple neurons. In such cases all spikes were regarded as one multi-unit response (MU). Data from single unit and multi-unit responses were pooled for analysis.

Units that had an autocorrelation >0.5 for either black or white had a clearly defined receptive field and were taken into the analysis. In a few cases the receptive field was on the border of the monitor (or ‘walked off the monitor’ with an increasing delay) and these neurons were excluded from analysis beforehand even for high autocorrelation scores.

For both white and black stimuli, we calculated the optimal latency at which the autocorrelation was highest. Since this latency was not always the same for white and black, we determined the size of the receptive field for white and black at their respective optimal latencies. The receptive field can be regarded as that area in which visual stimuli evoke activity above spontaneous activity, and this spontaneous activity must be accounted for. Therefore, the spontaneous activity was calculated by averaging the activity to the presentation of a white or black stimulus in each corner of the grid. We chose the corners since the grid was made larger than the estimated receptive field and thus the border grid units were always outside the receptive field (see receptive field approximation). If activity was higher than 25% of the maximum response (corrected for the spontaneous activity) it was regarded as part of the receptive field. Receptive field areas (in degrees squared) were determined by multiplying the number of grid pixels by the width and the height of each grid unit. The receptive field size in degrees was then calculated by taking the square root of the area.

In order to quantify the difference in the receptive field sizes with black and white stimuli, we calculated the contrast index, which is defined as the difference in receptive field size between the black and white subfields relative to their combined size: (black subfield size – white subfield size)/(black subfield size + white subfield size). Negative values represent neurons whose white subfield is larger, while positive values represent neurons whose black subfield is larger. Thus, extreme values near 1 or −1 indicate a large difference in subfield size for white and black, whereas small values (near 0) indicate no difference. In addition, we calculated the overlap between the white and the black subfield by calculating the percentage of the pixels of the smallest subfield that were also part of the larger subfield.

Correlations between the size of the black or white subfield and the depth of the recording were tested using Spearman's correlation coefficient. Differences in receptive field size and the maximum number of spikes were tested for significance with a MANOVA where contrast was regarded as a within-factor. A one-sample t-test was used to test whether the contrast index differed from zero.

## Results

In order to gain further insight into the processing of visual information in the optic tectum, we recorded the receptive fields of tectal neurons in the superficial, intermediate and deep layers. We successfully applied sparse noise stimuli commonly used to record receptive fields of neurons in the visual cortex to map the receptive fields of neurons in the optic tectum [26], [27], [28], [29]. With this method we could describe the receptive fields in the optic tectum with a high spatial and temporal precision (see figure 1).

### Description of the Receptive Field

We recorded 140 units from the superficial (depth<500 µm; 5 SU, 20 MU), intermediate (depth between 500–1200 µm; 23 SU, 27 MU) and deep layers (depth>1200 µm; 55 SU, 10 MU) in the optic tectum in anesthetized chickens.

The shape of the receptive field altered with increasing depth in the optic tectum. While the receptive fields of units encountered in the superficial layers were mostly round or oval-shaped (see figure 2), more irregular and complex shaped receptive fields were found for deeper located neurons (see figure 3 and 4).

**Figure 5:**
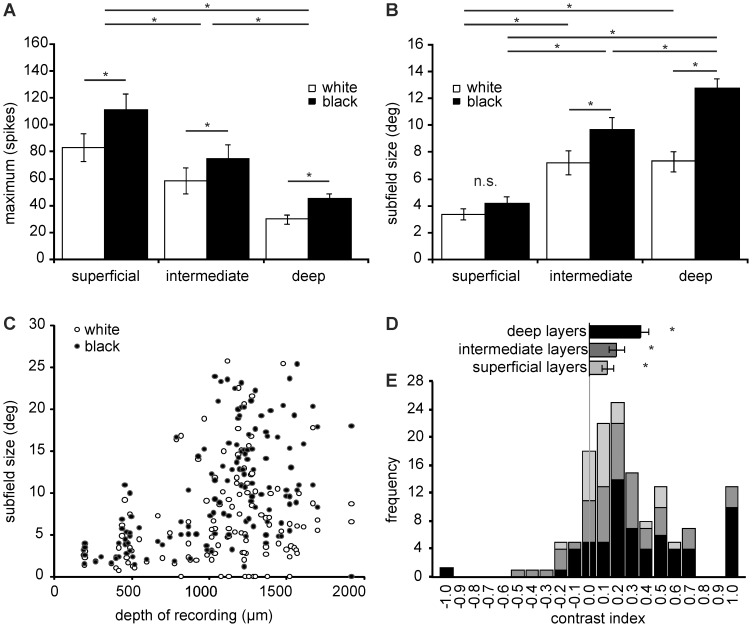
Receptive field properties. a) Maximum number of spikes in response to a black or white stimulus for the superficial layers, intermediate layers and deep layers. Responses to white stimuli are shown in white and responses to black stimuli in black. b) Average size of the black subfield and the white subfield for the superficial layers, intermediate layers and deep layers. Responses to white stimuli are shown in white and responses to black stimuli in black. c) Sizes of the black subfield and the white subfield in relation to the recording depth in the optic tectum. Open circles represent the white subfields while closed circles represent the black subfields. d) Average contrast index for the superficial (shown in light grey), intermediate (shown in dark grey) and deep layers (shown in black). Negative values represent neurons whose black subfield was larger, while positive values represent neurons whose white subfield was larger. e) Distribution of the contrast index values for the superficial layers (shown in light grey), intermediate layers (shown in dark grey) and deep layers (shown in black). Please notice that the majority of the neurons has a contrast index >0 indicating a larger subfield for black than for white. Error bars represent standard errors. *p<0.05.

Receptive fields recorded deeper in the tectum were larger (superficial layers 3.8 degrees, intermediate layers 8.5 degrees, deep layers 10.0 degrees; see figure 5). Especially, receptive fields in the intermediate (interaction between layer and contrast F_2,137_ = 7.536 p = 0.001; posthoc p<0.001) and deep layers (p<0.001) were larger than those in the superficial layers. Not surprisingly, the size of the subfields correlated with the depth of the recording for both white (Spearman correlation r = 0.424 p<0.001 n = 127) and black (Spearman correlation r = 0.567 p<0.001 n = 139; see figure 5c).

### Black and White Subfields

Overall, the maximum activity evoked by a single white or black stimulus was highest in the superficial layers and lowest in the deep layers (main effect of layer F_2,137_ = 13.549; superficial-intermediate p = 0.047, superficial-deep p<0.001; intermediate-deep p = 0.008; see figure 5a). Most units responded with a higher number of spikes to black than to white stimuli (main effect of contrast F_2,137_ = 70.137 p<0.001; see figure 5a). Accordingly, the average size of the black subfield was larger than the white subfield (see figure 5b). More importantly, this size difference was layer-dependent (interaction between layer and contrast F_2,137_ = 7.536 p = 0.001). For both the intermediate and deep layers, the difference in average receptive field size was statistically significant (intermediate p = 0.002, deep p<0.001). While the black subfield was also slightly larger than the white subfield for the superficial layer, this was not significant (p = 0.463).

This effect was observed for both single units and multi units (interaction SU/MU with contrast F1,134 = 5.334), with the effect being significant for single units (p<0.001). However, due to the small number of units, separate statistical analysis of single units and multi units along the different layers of the optic tectum was not possible, and the data had to be pooled.

In order to quantify the relative size of the black and white subfields, we calculated the contrast index. A negative index indicates that the white subfield is larger, while a positive index indicates that the black subfield is larger. An index of zero denotes that the subfields were exactly the same size.

Most units were found to have a larger black subfield resulting in a positive contrast index (see figure 5d–e). Although the difference between the black and the white subfield tended to increase with increasing depth, it was found to be significantly higher than zero for the superficial layers (t = 3.140, p = 0.004), intermediate layers (t = 3.763, p<0.001) as well as the deep layers (t = 6.680, p<0.001). In addition, 13 units were found to only have a black subfield and no white subfield. All of these units were found in the intermediate and deep layers (intermediate 3/50 or 6.0%, deep 10/65 or 15.4%). In contrast only 1 unit from deep layers was found to have a white subfield only (1/65 or 1.54%).

Often the white and black subfields showed some degree of overlap. The amount of overlap between the white and the black subfield decreased along the tectal layers with cells in the deeper part of the tectum generally showing less overlap in their receptive fields (data not shown).

### Response to Change in Luminance

It is reasonable to suppose that the recorded tectal neurons respond to a difference in contrast, i.e. to a perceived difference in luminance. In our experimental setup, relative to the grey background, the luminance increased the same amount after turning on a white dot or turning off a black dot. Similarly, the luminance decreased after turning off a white dot or turning on a black dot. If neurons would solely respond to such an increase or decrease in luminance, they would respond to both events, although at different moments in time. While the short latencies most likely represent responses to the onset of the stimulus, the responses at longer latencies probably represents responses to the offset of the stimulus. Often such responses to the onset and offset of the stimulus are seen as well in the classical Post-Stimulus Time Histogram (PSTH) whereby the response is plotted relative to stimulus onset. If a cell responds to a change in luminance one would expect a response to the onset of one contrast (either white or black) and to the offset of the other.

We found a total of 15 (out of 140) of such “luminance” neurons (see figure 3a). Thus, the delay at which the best response was evoked was short (roughly up to one stimulus duration, i.e. up to 50 or 100 ms) for a white stimulus and long for a black stimulus (i.e. longer than 50 or 100 ms), or vice versa. Of these 15 cells, 9 responded to onset of black and the offset of white (i.e. ‘decrease’), while 6 responded to onset of white and offset of black (i.e. ‘increase’). Such cells were found more or less evenly distributed in the superficial layers (1/25 or 4%), intermediate layers (6/50 or 12%) and the deep layers (8/55 or 15%) of the tectum (Chi-Square Χ2 = 1.437 p = 0.517).

### Responses to White and Black Bars

While searching for cells we noticed that often the neurons responded better to large black stimuli while white stimuli of the same size did not evoke any response. Often we were only able to evoke responses using white stimuli after decreasing the stimulus size. Therefore, we decided to test a subset of the cells with both black and white bars moving through the receptive field.

**Figure 6:**
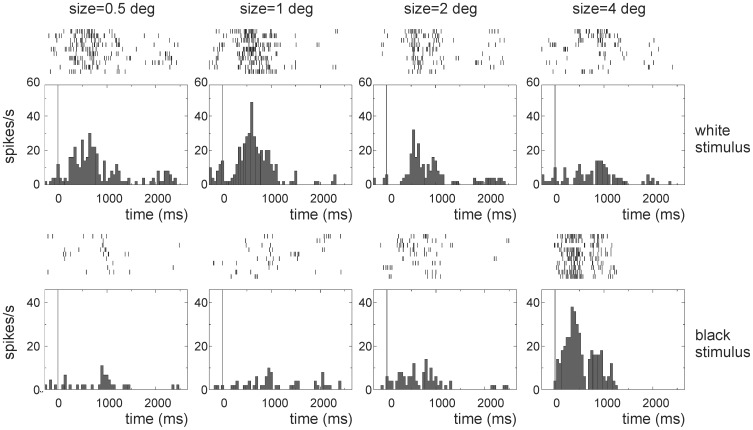
Example of the response to a white and a black bar. PSTH and raster plot of a single unit (recorded at 1700 µm depth) in response to white and black bars swept through the receptive field in the preferred direction. The upper row shows the responses to a 0.5, 1, 2 or 4 degrees wide white bar while the lower row shows the responses to a similar-sized black bar. This neuron responds almost as strong to a small white stimulus as to a large black stimulus.

In total, we tested 20 units (4 from the superficial layers, 6 from the intermediate layers and 10 from the deep layers) with white and black bars moving through the receptive field. Interestingly, in all 20 cases the black stimulus that evoked the best response was a large 4 degrees bar, the largest stimulus used. With a white bar (0.5–4 degrees), a good response was only evoked in 13/20 cases, and in the far majority of the cases the best size for a white stimulus was much smaller than 4 degrees. In 10/13 cases a white stimulus of 1 degree or smaller gave the best response (see figure 6). In those cases where a good response was observed with both black and white bars, the majority (9/13) responded with a higher number of spikes to the black bar than to the white bar.

## Discussion

We recorded the receptive fields of neurons in the optic tectum of chickens using sparse noise, where white and black stimuli are shown in fast progression without inter-trial interval. By reversely correlating the occurrence of each spike with the black or white stimulus presented up to a few hundred ms before, the receptive field was obtained in high spatial precision.

In agreement with previous studies, we found that receptive field sizes increase from the superficial layers to the deeper layers [9], [12], [18]. Moreover, receptive field shapes often were more complex in the deeper parts of the tectum [18], [20], [21], [23].

Most interestingly, our results consistently showed that the black subfield is larger than the white subfield, which is sometimes not present at all. This difference between black and white was demonstrated for both flashing dot and moving bar stimuli, and increased along the depth of the tectum. In addition, it was shown that the best white stimulus was a very small bar while the best black stimulus was a large bar.

### Stronger Responses to Black Stimuli

The observation that the majority of the neurons recorded from the optic tectum responded far better to black stimuli than to white stimuli is at least peculiar. Luminance differences can be ruled out as a reason since we kept the luminance difference between white and grey the same as between black and grey. If the stronger responses to black were indeed caused by a difference in luminance, we would expect to find a strong preference for either white or black in all layers. Our results, however, show a different pattern; while we found larger black subfields for intermediate and deep layers, subfields for white and black did not differ significantly for the superficial layers. In addition, neurons responding exclusively to black stimuli were only found in the intermediate and deep layers, but never in the superficial layers.

Moreover we found a small number of cells in the tectum that responded to a general increase or decrease in luminance. Thus, the onset of a white stimulus evoked a similar response as the offset of the black stimulus or vice versa, which indicates that these cells respond to the increase or decrease in luminance. However, such cells formed only a small minority of the recorded cells.

Using flashing stimuli, we found that many neurons in the tectum responded differently to white compared to black stimuli, with the latter often evoking stronger responses. It was shown before that many neurons cannot be mapped using white dots on a black background [12]. Interestingly, Gu et al [22] found that those cells that could not be mapped with flashing white stimuli responded almost equally to both black and white moving stimuli. Moreover, Hughes et al [18] showed that cells in the deeper layers were less likely to respond to light flashes than cells in the superficial layers, which coincides with our finding that some neurons responded exclusively to black stimuli in the intermediate (3/50) and deeper layers (10/65). Alternatively, the absence of responses to white could be due to stimulus sizes being too large (i.e., larger than the excitatory center for white). Frost et al. [14] found that tectal neurons cease their response if their surround is stimulated. Experiments with bar stimuli demonstrated that neurons with a classical inhibitory surround decrease their response when the stimulus is larger than optimal [11], [36].

Often the white subfield 'fitted into' the black subfield. Such an organization of the receptive field was observed more often for neurons in the intermediate and deep layers than for the superficial layers. This raises the question whether the receptive fields of these neurons could have a center-surround organization [37] whereby the center responds to white stimuli and the surround to black stimuli. Nevertheless we would then expect a ‘hole’ in the black subfield at the position of the white subfield, something we never observed. Moreover, the receptive fields of tectal neurons that showed a small white subfield located within a larger black subfield organization were often very complex in shape, far from being concentric (see figure 4 for examples).

In order to investigate the receptive fields of tectal neurons in more detail, we stimulated a small subset of neurons with white and black bar stimuli. Although responses could be evoked with a range of moving stimuli, we noticed an interesting difference when using white and black moving bars. In most cases cells responded best to smallest white stimuli used (0.5–1 degrees) while responding best to the largest black bar stimulus used (4 degrees). Thus, the excitatory center was very small when recorded with white stimuli while it was much larger when recorded with black moving bars, in agreement with our results for flashing stimuli.

A subpopulation of neurons in the deeper layers has been reported to have spotty receptive fields that consist of a central hotspot responding to both moving and flashed stimuli and several movement-selective subfields [20], [21]. These neurons have been shown to respond best to very small spots of light [20], [21], which would well agree with a preference for small stimuli as we found in the neurons we tested with white and black moving bars. It is thus possible that, for some cells, the seemingly small receptive subfield for white is only the hotspot of a larger receptive field. Since these cells are thought to be involved in the processing of motion stimuli [6], [38], [39] and contain movement sensitive subfields, it would thus not be surprising that we did not find a large receptive field using our flashed white stimuli.

### Magnification of Black Responses along the Tectal Layers

Our results are surprisingly similar to results in the visual cortex of mammals measured using the same technique. While for the input layer of the visual cortex (layer 4c), neurons were found to respond equally to black and white with only a slight preference to black, the output layers (layer 2/3) showed a striking preference for black stimuli [40], [41]. Interestingly, our recordings from the optic tectum show a similar pattern; the superficial (input) layers respond roughly equally to black and white stimuli, while the intermediate and deep (output) layers respond much stronger to black stimuli. Although we cannot draw strong conclusions regarding the underlying mechanisms based on our dataset we hypothesize that this is due to recurrent loops within the tectum, similar to the visual cortex [41]. An alternative explanation is that the increasing response to black stimuli results from descending projections of the visual Wulst, the avian homologue of the visual cortex [42]. The Wulst is known to project onto the tectum [43], [44], [45] and can influence tectal responses [46]. As the Wulst has been found to contain neurons which respond to a black bar only [47], this might add to the preponderance of black in the tectum.

We can only speculate on the evolutionary advantage for an amplification of signals to black stimuli. Our recordings were restricted to neurons in the accessible tectum [5], which receive information from the upper lateral part of the visual field when the animal stands straight up. It is tempting to hypothesize that such a bias might have evolved to better see a black object against a brighter background (i.e. possible predator in the sky).

Nevertheless this still leaves the question open why cells respond best to small white stimuli and large black stimuli. While we do not have an immediate answer to this question we speculate that the responses to small white stimuli are involved extracting fine spatial location [14], [18], [48], [49], which could be necessary for accurate localization of small particles of food, whereas relevant dark stimuli such as predators in the sky would be larger. Future experiments will have to determine whether the part of the tectum that receives input from the area dorsalis of the retina, which is relevant for closer inspection, feeding behavior and the control of pecking [50] has a similar bias towards black stimuli.
